# Oral lichen-planus-associated fibroblasts acquire myofibroblast characteristics and secrete pro-inflammatory cytokines in response to Porphyromonas gingivalis lipopolysaccharide stimulation

**DOI:** 10.1186/s12903-018-0656-6

**Published:** 2018-11-29

**Authors:** Liping Wang, Yinshen Yang, Xiaoqin Xiong, Ting Yu, Xinhong Wang, Wenxia Meng, Haiyan Wang, Gang Luo, Linhu Ge

**Affiliations:** 1 Key Laboratory of Oral Medicine, Guangzhou Institute of Oral Disease, Stomatology Hospital of Guangzhou Medical University, Guangzhou, 510140 China; 2 Department of Oral Medicine, Stomatological Hospital, Southern Medical University, Guangzhou, 510220 China

**Keywords:** Oral lichen planus, Myofibroblast, Interleukins

## Abstract

**Background:**

Oral lichen planus (OLP) is a chronic inflammatory oral mucosal disease in which comprehensive inflammation-related cytokines are involved. These cytokines are commonly produced by immune cells and specific nonimmune cells including keratinocytes, endothelial cells and fibroblasts. This raises the question of whether fibroblasts in OLP lesions contribute to the inflammatory process upon inflammatory simulation.

**Methods:**

Primary cultured Oral lichen-planus-associated fibroblasts (OLP AFs, *n* = 5) and normal buccal mucosal fibroblasts (NFs, *n* = 5) were examined by immunohistochemistry, Western blotting and reverse transcription-polymerase chain reactions (RT-PCR). Various inflammatory mediators were evaluated with a multiplex assay. Differences among groups were assessed using a Student’s test or repeated measures one-way ANOVA, as appropriate.

**Results:**

OLP AFs express significantly higher levels of α-smooth muscle actin (α-SMA) than NFs, indicating the presence of myofibroblasts. Myofibroblasts secrete Interleukin (IL)-6, IL-8, and tumor necrosis factor-α (TNF-α) in response to Porphyromonas gingivalis lipopolysaccharide (pg. LPS).

**Conclusion:**

OLP AFs demonstrated α-SMA expression and secreted pro-inflammatory cytokines in response to pg. LPS stimulation.

**Electronic supplementary material:**

The online version of this article (10.1186/s12903-018-0656-6) contains supplementary material, which is available to authorized users.

## Background

Oral lichen planus (OLP) is a chronic inflammatory oral mucosal disease with the potential for malignant transformation [1]. The prevalence of OLP varies from 0.5 to 4% in the general population [2], and the malignant transformation rate is 0–2%. The clinical presentation of OLP involves a ranges from asymptomatic white keratotic lesions to painful erosions and ulcerations, with three distinctive clinical forms: reticular, erosive, and atrophic. Up to now, diagnosis of malignant transformation have been performed with biopsies. The histology of OLP is characterized by a dense subepithelial lympho-histiocytic infiltrate, increased numbers of intra-epithelial lymphocytes, and degeneration of basal keratinocytes.Degenerating basal keratinocytes form colloid bodies that appear as homogenous eosinophilic globules [1] In retrospective studies of Chinese cases, the erosive form was the most likely type to develop into oral squamous cell carcinoma (OSCC) [3–5]. Additionally, increased levels of interleukin (IL)-1, IL-6, IL-4, tumor necrosis factor-α (TNF-α), interferon-γ (IFN-γ) and other cytokines are more closely associated with the erosive form than the reticular and atrophic forms [6–8]. Although the pathogenesis is not completely clear, immune and inflammatory factors are thought to play crucial roles in the development of OLP [9]. Aberrant production of many inflammatory mediators occurs in OLP lesions. Cytokines and chemokines, including ILs [10], transforming growth factor-β (TGF-β), IFN-γ and TNF-α [11–13], have been strongly implicated.

In the environment of a dysregulated network of cytokines, fibroblasts are likely to be transformed into myofibroblasts, and the central feature of these cells in the activated state is the acquisition of α-smooth muscle fibrin [14, 15]. In healthy mucosa, MFs are absent from the epithelial and stromal cell populations. In OSCC, the stroma contains MFs among the malignant epithelial cells, which regulate the proliferation and apoptosis of the epithelia. In advanced oral submucous fibrosis (OSF), MFs are subjacent to the epithelium and secrete collagen I [16, 17]. In cyclosporine A-induced gingival overgrowth and hereditary gingival fibromatosis of connective tissue, intense α-SMA staining is observed, and MFs contribute to fibrosis by secreting several inflammatory cytokines [18, 19]. MFs are a rich source of chemokines, cytokines, and extracellular matrix (ECM) proteins, which are involved in neoplasms and some inflammatory processes. The dynamic role of MFs in OSCC, OLK and OSF has been reported in previous studies [10]. However, no evidence exists of a relationship between MFs and OLP.

The original morphologic characterization of myofibroblasts by electron microscopy showed them to have numerous well-formed cytoskeletal microfilaments. These microfilaments were subsequently found to be composed of actin filaments and smooth muscle “specific” actin. The presence of actin stress fibers was similar to that seen in smooth muscle cells, but these cells also retained typical fibroblast features [20].

A large body of evidence suggests that gingival fibroblasts can react to various stimuli by releasing cytokines and chemokines, which ultimately play important roles in the inflammatory response [21].

We hypothesized that OLP fibroblasts are transformed into myofibroblasts and contribute to the inflammatory process in OLP lesions.

## Materials and methods

### Specimens

The protocol was approved by the Ethics Committee of the Stomatology Hospital of Guangzhou Medical University, (No. KY2017019, Guangzhou 510,140, China). Informed consent was obtained from each patient. Sections of human mucosa (*n* = 5) were obtained from mucosa discarded during biopsies. Normal healthy mucosa was obtained from samples discarded during plastic surgery at the Stomatology Hospital of Guangzhou Medical University.

Patients were eligible for inclusion if they (1) were between 18 and 50 years old and (2) had a diagnosis consistent with erosive OLP without epithelial dysplasia. Patients were excluded if they (1) had severe systemic diseases, (2) received antibiotics or other medicines in the previous 4 weeks, or (3) had other oral mucosal diseases. Patient information for the OLP and normal specimens is shown in Table 1.

**Table 1:** Details of the specimens

Case	Gender	Age (year)	Site of biopsy	Clinical form
1	Female	32	Buccal	Erosive form
2	Female	28	Buccal	Erosive form
3	Male	28	Buccal	Erosive form
4	Male	24	Buccal	Erosive form
5	Female	41	Buccal	Erosive form

### Primary culture

Freshly resected specimens were washed with sterile phosphate-buffered saline (PBS) (BOSTER, AR0030, CA) containing 10% penicillin-streptomycin-glutamine (100X) (Gibco, 10,378,016, Thermo Fisher Scientific Inc.) before being minced into 1-mm^3^ fragments. These fragments were cultured in DMEM medium (Gibco, Thermo Fisher Scientific Inc., Waltham, MA, USA) supplemented with 10% fetal bovine serum (10,099,141, Gibco) at 37 °C with 5% CO_2_. This medium was used for carcinoma-associated fibroblasts (CAFs) and NFs [22–24]. Cultured cells at passages 3–6 were used.

### Scanning electron microscopy (SEM)

The samples were rinsed with PBS and fixed overnight in 3% glutaraldehyde at 4 °C. Next, the samples were dehydrated using the following ethanol gradient: 30, 50, 70, 95, and 100%. Then, the samples were dehydrated with xylene and air-dried at room temperature. Subsequently, the specimens were coated with gold and examined by SEM (HITACHI-S-3400 N, Hitachi High-Technologies Corporation, Tokyo, Japan). The obtained images were processed using Adobe Photoshop CS6 software (Adobe Systems Inc., San Jose, CA, USA).

### Immunohistochemistry and immunocytochemistry

Immunohistochemistry: Mucosal sections were processed for histology. The Diva Decloaker reagent was used for antigen retrieval in formalin-fixed, paraffin-embedded tissues. α-SMA and vimentin were detected with monoclonal rabbit anti-α-SMA (ab32575, Abcam, Cambridge, MA) (1:300) and anti-vimentin (ab92547, Abcam) (1:300), followed by treatment with goat anti-rabbit IgG H&L (HRP, ab6721, Abcam) and diaminobenzidine.

Immunocytochemistry: Primary cells were seeded on glass coverslips in six-well plates. At least five randomly selected visual fields of the immunostained cells were examined under a light microscope (Leica, DM4000, Tokyo, Japan). The obtained images were compiled using Adobe Photoshop CS6 software.

Immunofluorescence staining was performed on paraffin-embedded sections of normal and diseased human oral mucosal specimens. Rabbit polyclonal antibodies to CD31 (ab28364, Abcam) (1:25) or mouse monoclonal antibodies to α-SMA (ab7817, Abcam) (1:200) were applied overnight, followed by incubation with goat anti-rabbit Cy2 (111–225-144, Jackson ImmunoResearch, West Grove, PA) or a mouse monoclonal antibody to α-SMA (ab205719, Abcam) (1:250), respectively. Cells were examined with a fluorescence (Leica, DM4000, Instruments, Melville, NY) or confocal (Leica, tcssp8, NY) microscope.

### Reverse transcription-polymerase chain reaction (RT-PCR)

RNA was harvested from primary cultures established from five different buccal mucosa samples using the TRIzol reagent (Invitrogen Life Technologies) according to the manufacturer’s protocol and reverse transcribed into first-strand cDNA with the gDNA eraser (RR047A, TAKARA) transcription system. RT-PCR was performed with SYBR® Premix Ex Taq™ II (TAKARA, RR820A) using a Bio-Rad system, and GAPDH and β-actin were used as internal controls. Gene expression was quantified relative to the internal controls. All primers are listed in Table 2. The experiments were repeated a total of three times.

**Table 2:** Primer sequences of the investigated genes

Gene	Forward primer (5′- > 3′)	Reverse primer (5′- > 3′)	Annealing temperature (°C)	Product length (bp)
α-SMA	TGGGGAAAGTAGATCGGACAG	AACAGTGGAAAGTTGGGGACA	60	164
Vimentin	TTGTTCTCCAGGTACAGGTTACTA	GAGGACATCAACACCCAAATC	60	89
GAPDH	GCGATACTCACTCTTCTACCTTCGA	TCGTACCAGGAAATGAGCTTGAC	60	82

### Western blotting

Primary cells were lysed with RIPA buffer and a proteinase inhibitor mixture (PMSF). Nuclear and cytoplasmic proteins were separated using a nuclear and cytoplasm protein extraction kit (78,833, Thermo Pierce, Rockford, IL, USA), according to the manufacturer’s instructions. The protein concentration was assessed using a Multiskan instrument (Thermo, Multiskan Mk3). Total proteins were electrophoresed on 12% SDS-PAGE gels and transferred onto polyscreen PVDF transfer membranes (Immobilon-P PVDF Membrane, Merck Millipore). Membranes were blocked with 5% (*w*/*v*) nonfat milk in Tris-buffered saline (TBS) containing 0.1% Tween 20 for 1 h at room temperature and incubated overnight with primary anti-α-SMA (ab32575, Abcam) (1:5000), anti-vimentin (ab92547, Abcam) (1:5000), anti-TLR2 (ab16894, Abcam) (1:1000) and anti-TLR4 (ab22048, Abcam) (1:1000) at 4 °C. After washing, an HRP-conjugated secondary antibody was added for 1 h at 37 °C. Detection was performed with enhanced chemiluminescence (ECL), and blots were quantified by densitometry using the accompanying computerized image analysis program (Image Tool).

### Lipopolysaccharide (LPS) stimulation

Cells grown in 12-well plates (5.0 × 10^4^ cells/well) were treated with the Toll-like receptor ligand pg. LPS (Invivogen) at a concentration of 10 ng/ml for 0, 2, 4, 8, 12, 24 or 48 h, and the corresponding supernatants were collected. PBS was used as a control.

### Human 9-plex assay

Conditioned media samples of LPS-treated cells were submitted in triplicate for Human 9-Plex assay testing to the USC Beckman Center Immune Monitoring Core Lab. Secreted cytokines and chemokines were analyzed with the Bio-Plex suspension array system (Bio-Rad Laboratories, Hercules, CA) using a Q-Plex Human 9-Plex Kit (110449HU, Quansys Biosciences, USA) to evaluate IL-1α, IL-1β, IL-2, IL-4, IL-6, IL-8, IL-10, IFN-γ, and TNF-α concentrations. Experiments were repeated a total of three times.

### Enzyme-linked immunosorbent assay (ELISA) and cytokine assay

Conditioned media samples from treated cells were collected, passed through a 0.45-μm filter to remove cell debris, aliquoted, and frozen at − 20 °C. An ELISA Kit (Abcam, ab46042, UK) was used to evaluate IL-6 concentrations at 0, 2, 4, 8, 12, 24 and 48 h, according to the manufacturer’s instructions. Cytokine levels in each sample were determined based on a standard curve generated by the recombinant proteins provided with the kits.

### Statistical analysis

The data were analyzed using Prism statistical software (GraphPad Software, La Jolla, CA). Differences among groups were assessed using a two-sided Student’s test or repeated measures one-way ANOVA, as appropriate. The results are expressed as means±SEM. *** *P* < 0.01; ** 0.01 ≤ *P* < 0.05; and NS *P* ≥ 0.05.

## Results

### OLP buccal mucosa contains stromal cells expressing α-SMA

**Fig. 1:**
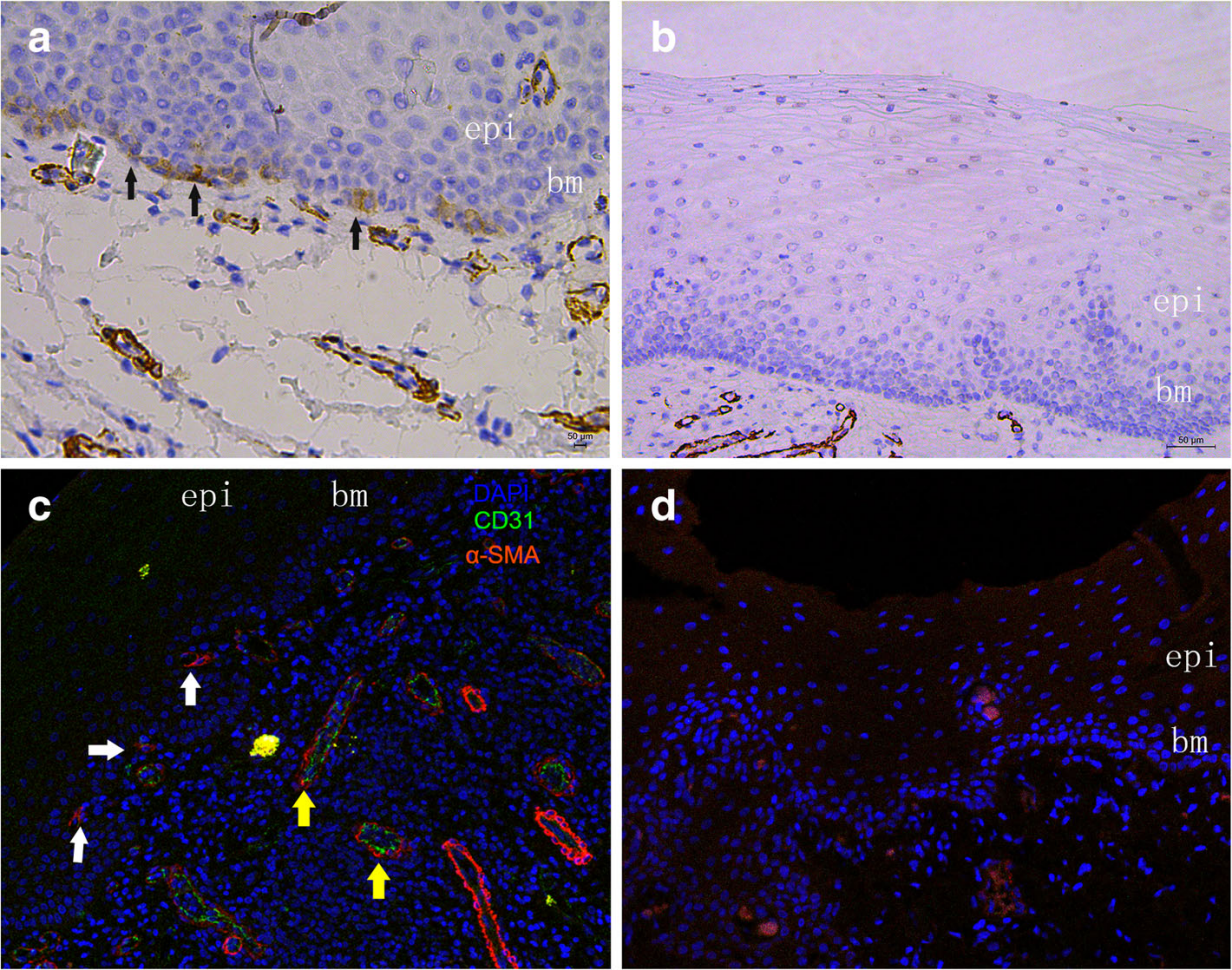
Immunohistochemical and Immunostaining for α-SMA in OLP and oral normal buccle tissue (NBT). α-SMA-positive stromal cells (black arrows) located in the subepithelial layer showing cytoplasmatic immunostaining (**a**) were considered as a marker of myofibroblasts. α-SMA(+)CD31(−) spindle-shaped cells(white arrows) around bm consisted with myofibroblast’s identification.Meanwhile, α-SMA(+)CD31(+) vascular endothelial cell(yellow arrows) formed as vessels (**c**) α-SMA staining was not observed in NBT subepithelium (**b** and **d**) (20-fold magnification). epi = epithelial. bm = basement membrane

Immunostaining demonstrated expression of α-SMA in spindle-shaped cells adjacent to the OLP mucosal epithelium (Fig. 1a), which was not found in normal buccal mucosa (Fig. 1b). α-SMA expression was also observed in endothelial cells forming blood vessels.

To determine whether the α-SMA expression was in MFs or endothelial cells, we performed immunostaining for α-SMA and CD31. Each DAPI-stained nucleus was evaluated for expression of α-SMA and CD31. Confocal microscopy demonstrated that in OLP mucosa, spindle-shaped cells around the basement membrane occasionally expressed the MF marker α-SMA (Fig. 1c). Endothelial cells forming blood vessels were also α-SMA+ and were distinguished from spindle-shaped cells by their circular configuration and the specific endothelial marker CD31. Immunofluorescence of normal mucosa did not show α-SMA staining near the squamous epithelium (Fig. 1d).

### Primary cultured OLP AFs express α-SMA in vitro

**Fig. 2:**
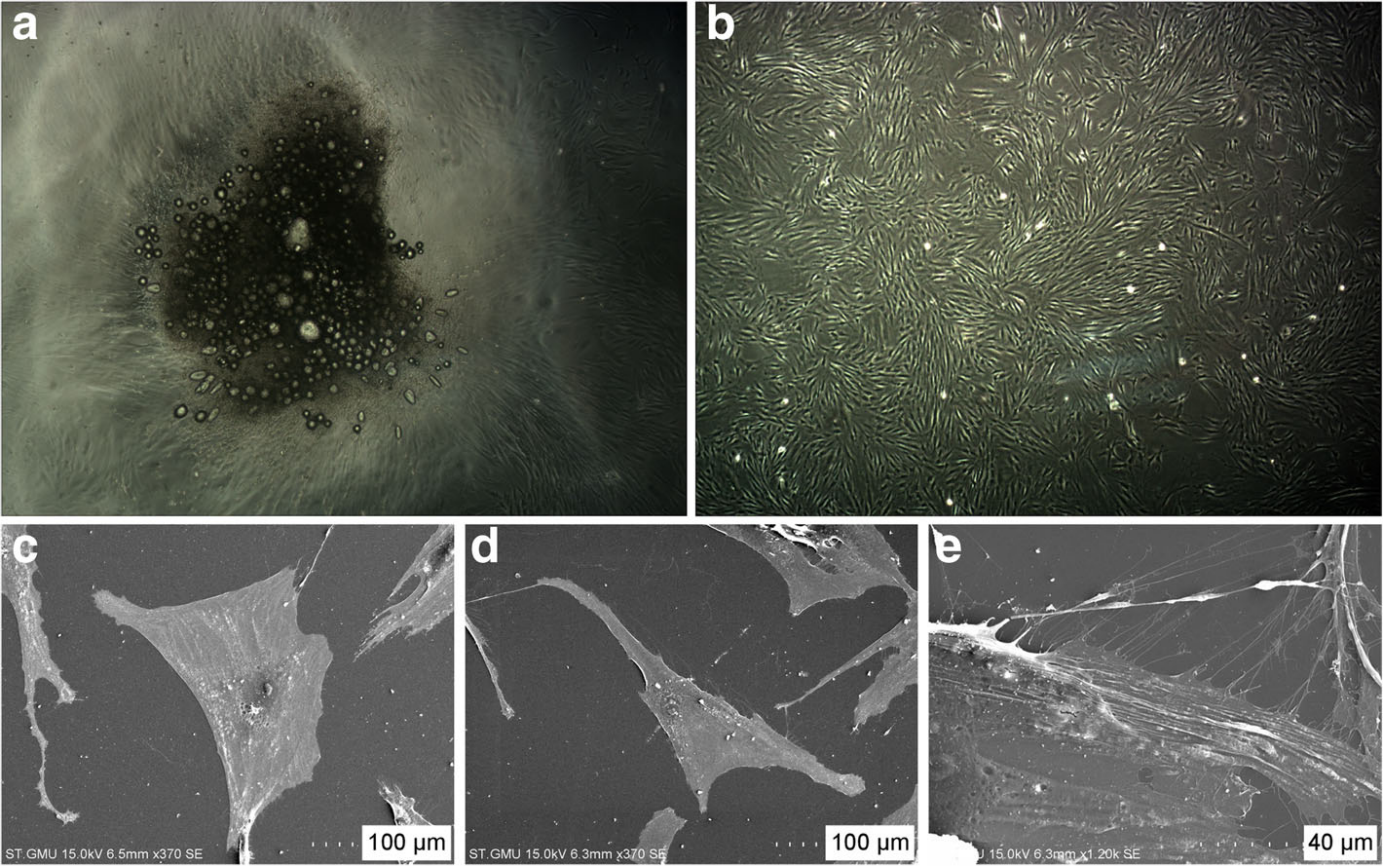
Within 7–21 days, the mixed cell suspension flattens out and adheres to the plate bottom, followed by an outgrowth of spindle-shaped cells (**a** and **b**). Scanning electron microscopic of the primary culture. Large, polygonal shape of AF (**c**) with bundles of fibrils resembling those of smooth muscle arranged parallel to the axis of the cell in the cytoplasm (**e**), and long and thin spindle shape of NF (**d**) can be observed

After plating, the heterogeneous cells adhered to the bottom of the plate (Fig. 2a). Adherent mucosal stromal cells retained a spindle-shaped morphology (Fig. 2b).

Proliferating cells from the OLP region were large and polygonal in shape (Fig. 2c) and the cells from NF group present long and thin spindle shape (Fig. 2d). Bundles of packed fibrils resembling smooth muscle developed within the cytoplasm and were usually arranged parallel to the axis of the cell (Fig. 2e).

**Fig. 3:**
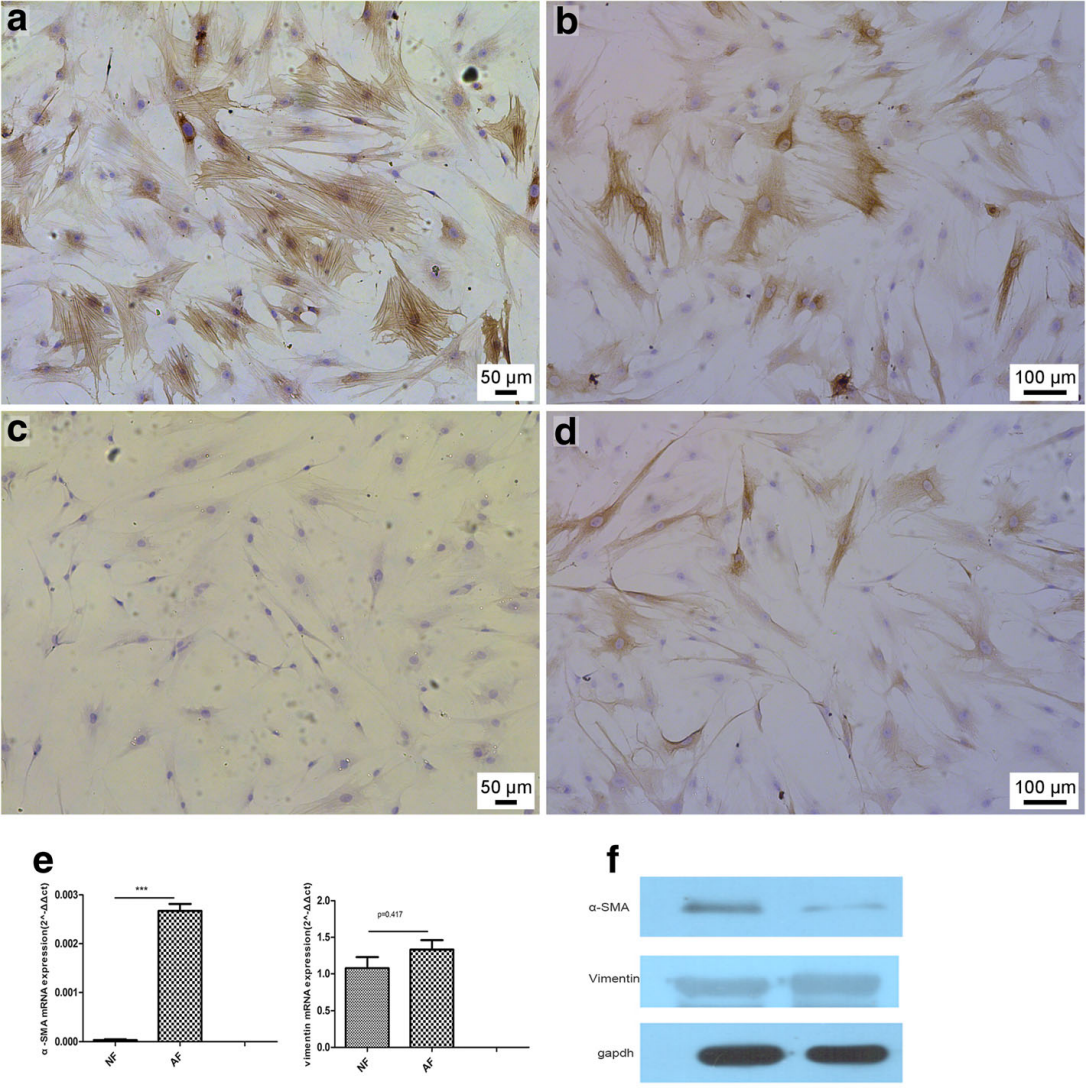
OLP mucosal stromal cells in primary culture coexpress α-SMA and vimentin (**a** and **b**). Primary cultured cells from normal mucosa lacked α-SMA expression but expressed vimentin (**c** and **d**). Expression of α-SMA and vimentin mRNA harvested from AF and NF was evaluated by quantitative RT-PCR. mRNA expression of α-SMA from AF was significantly higher than that of NF. Vimentin expression showed no significant difference between the groups (*p* = 0.471) (**e**). Western blot analysis performed with total cellular proteins from NF and AF clones. AF expressed high levels of α-SMA, whereas fibroblast clones lacked expression. NF and AF showed no significant difference in vimentin expression. GAPDH was used as an internal control (**f**)

Immunohistochemical staining for intracellular cytoskeletal proteins demonstrated that OLP mucosal stromal cells in primary culture coexpressed α-SMA and vimentin (Fig. 3a and b); primary cultured cells from normal mucosa lacked α-SMA but expressed vimentin (Fig. 3c and d).

The relative mRNA expression of α-SMA in OLP AFs was significantly higher than in NFs (*** *P* < 0.01), whereas no significant difference in vimentin was observed between the OLP AFs and NFs (*P* = 0.221) (Fig. 3e) Immunoblot suggested that AFs express higher α-SMA levels than NFs (Fig. 3f).

### Increased secretion of inflammation-related cytokines by OLP AFs in response to LPS stimulation

**Fig. 4:**
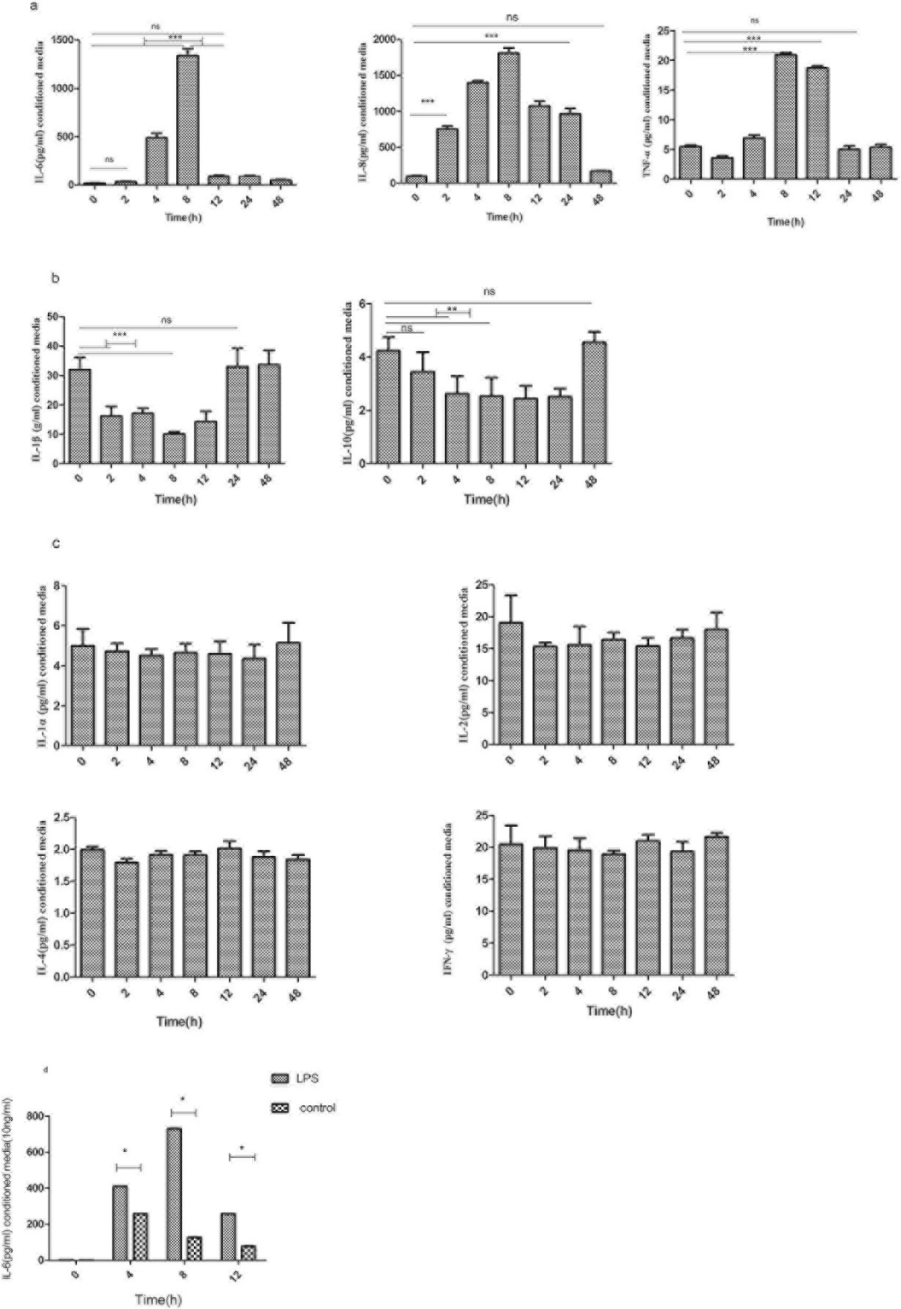
AFs were stimulated with LPS (10 ng/ml) for 0, 2, 4, 8, 12, 24 and 48 h, and supernatant was harvested for 9-plex cytokine evaluation (**a**-**c**). IL-6 was detected in OLP AF- and control-conditioned media using ELISA (**d**)

Human 9-Plex cytokine/chemokine screening demonstrated a continuous increase in the secretion of IL-6, IL-8, and TNF-α from 0 to 8 h after pg. LPS stimulation (Fig. 4a). The expression began to significantly decline at 12 h, and no difference was found at the 48-h time point compared to 0 h. Statistically significant decreases in IL-1β and IL-10 were detectable after 2 and 4 h (Fig. 4b). No significant differences were detected in IL-1-α, IL-2, IL-4, and IFN-γ at any timepoint in response to pg. LPS stimulation (Fig. 4c).

IL-6 was detected in OLP AF- and control-conditioned media using ELISA. IL-6 was continuously increased, and significant differences in IL-6 were observed between OLP AF- and control-conditioned media at 4, 8, and 12 h (Fig. 4d).

Four hours after LPS stimulation, we observed a significant increase in IL-6 in OLP AF-conditioned media (Table 3). Maximal IL-6 expression occurred after 8 h, with a nearly 250-fold increase compared to the level at 0 h, which began to significantly decline at 12 h compared to the concentration at 4 h.

**Table 3:** Relative chemokine expression of AFs and NFs in response to pg. lipopolysaccharide stimulation (AF=oral lichen-planus-associated fibroblasts, NF=fibroblasts)

	0	4	8	12	24	48
AF	3.1 ± 0.03	410.6 ± 0.07	731.5 ± 0.70	258.3 ± 0.03	55.8 ± 0.83	6.8 ± 0.73
NF	3.7 ± 0.11	85.0 ± 7.29	123.6 ± 2.26	140.1 ± 9.76	48.1 ± 3.05	6.8 ± 0.56
*P* value	0.0105	0.0005	< 0.0001	0.0003	0.0709	1.0000

lipopolysaccharide stimulation (AF = oral lichen-planus-associated fibroblasts, NF = fibroblasts)

## Discussion

OLP is a chronic inflammatory condition that involves the oral mucosal stratified squamous epithelium and the underlying lamina propria. The prevailing theories of OLP pathogenesis revolve around dysregulated T cell responses to exogenous triggers vs. a dysregulated autoimmune response to autologous keratinocyte antigens [25].

Nonspecific inflammation is a type of chronic inflammation that exhibits three main characteristics: (1) inflammatory cell infiltration; (2) tissue destruction; and (3) a repair response [26]. We consider OLP to be a model of nonspecific inflammation due to the following pathological and micrographic features: (1) liquefaction and degeneration of basal epithelial cells and band infiltration of superficial lymphocytes in the lamina propria [27]; (2) inflammatory cytokines, such as TNF-α, and MMPs that potentially cause a disordered arrangement of epithelial basal cells and the fracture of junctions between the stratum basale and basement membrane [28]; and (3) an increased number of Langerhans cells and mast cells in OLP lesions [29]. Although a large number of literatures have shown that pro-inflammatory cytokines are increased in OLP lesions, saliva and serum, its mechanism is still unclear.

Myofibroblasts are a highly specialized set of differentiated cells that play a prominent role during the body’s response to injury but can also contribute pathologically to inflammatory conditions such as fibrosis and cancer [30]. A key feature of myofibroblasts is expression of α-SMA.With improved diagnostics, lineage tracing studies, and genetic tools, a number of myofibroblast precursors have been identified, including fibroblasts, epithelial and endothelial cells via epithelial-to-mesenchymal transition (EMT) and endothelial-to-mesenchymal transition (EndoMT), resident mesenchymal progenitor cells,adipose tissue cells, and bone-marrow-derived circulating fibrocytes . [31] In previous studies, immunohistochemical observations demonstrated no expression of α-SMA in the epithelium and subepithelial tissue of and OLP mucosa, whether there are MFs located in OLP lesion is controversial.We immunostained the protein maker α-SMA and find that OLP buccal mucosa contains stromal cells expressing α-SMA.To identify these stromal cells, Immunohistochemical staining was used combined with observation of the morphology of the isolated cells.

Recently, several studies have suggested that microorganisms may trigger or be responsible for sustaining or exacerbating the chronic course of OLP [32–34]. Epithelial barrier dysfunction may precede intracellular infection of basal epithelial cells with bacteria, virus, or possibly fungus. In contrast to patients with oral mucosa and periodontal health, the expression of periodontitis related bacteria including porphyromonas gingivalis increased in the saliva of OLP patients and was positively correlated with the severity score.So We treated cells by LPS from P. gingivalis and Elisa to detect the Soluble cytokines in the collected supernatant. We found that LPS from P. gingivalis contributes to the over-production of pro-inflammatory cytokines in vitro, which may represent new evidence for the potential roles of microbial factors in the pathogenesis of OLP.

ILs are a group of cytokines with multiple roles in nearly all aspects of inflammation and immunity, including immune cell proliferation, differentiation, maturation and activation [35]. IL-6, IL8 and TNF-α are mainly secreted by antigen-presenting cells, including dendritic cells, macrophages and B cells, but are also expressed by a variety of nonimmune cells. Increases in IL-6, IL8 and TNF-α expression have been reported previously in OLP lesions and serum, especially the erosive forms [8].

There is no credible evidence can prove that the fibroblasts from OLP secret pro-inflammatory cytokines.According to our results,fibroblast from the normal buccal secret feebler pro-inflammatory cytokines than myofibroblast. In the model of microbial infection established in vitro, what cellular mechanism of pg.lps induces MFB to produce and release pro-inflammatory cytokines is be worth searching.

## Conclusion

1. A subset of fibroblasts in OLP lesions are transformed into myofibroblasts.
2. OLP AFs secrete pro-inflammatory cytokines in response to microorganismal infection.

## Additional files

Additional file 1: The detected concentrations of IL1-α, IL1-β, IL-2, IL-4, IL-6, IL-8, IL-10 IFN-γ and TNF-α from AF- and NF-conditioned media at the 0, 2, 4, 6, 8, 12, 24, and 48 h time points. (CSV 15 kb)

Additional file 2: Immunoblot of TLR2 and TLR4 expressed by AFs according to the time point. (TIF 266 kb)

Additional file 3: Immunofluorescence of IL-6, TLR2, and TLR4 expressed by AFs after 8 h of pg.LPS stimulation. (TIF 274 kb)

## Abbreviations

DMEM: Dulbecco’s modified Eagle’s medium
FBS: Fetal bovine serum
IFN-γ: interferon-γ
IL: Interleukin
LPS: Lipopolysaccharide
Pg.: Porphyromonas gingivalis
TLR: Toll-like receptor
TNF-α: tumor necrosis factor alpha
TNF-α: tumor necrosis factor-α
α-SMA: α-smooth muscle actin
