# Supplementary figures and images for: Oral lichen-planus-associated fibroblasts acquire myofibroblast characteristics and secrete pro-inflammatory cytokines in response to Porphyromonas gingivalis lipopolysaccharide stimulation
Source: BMC Oral Health. 2018 Nov 29;18:197. doi: 10.1186/s12903-018-0656-6 (PMC6267065; doi:10.1186/s12903-018-0656-6)

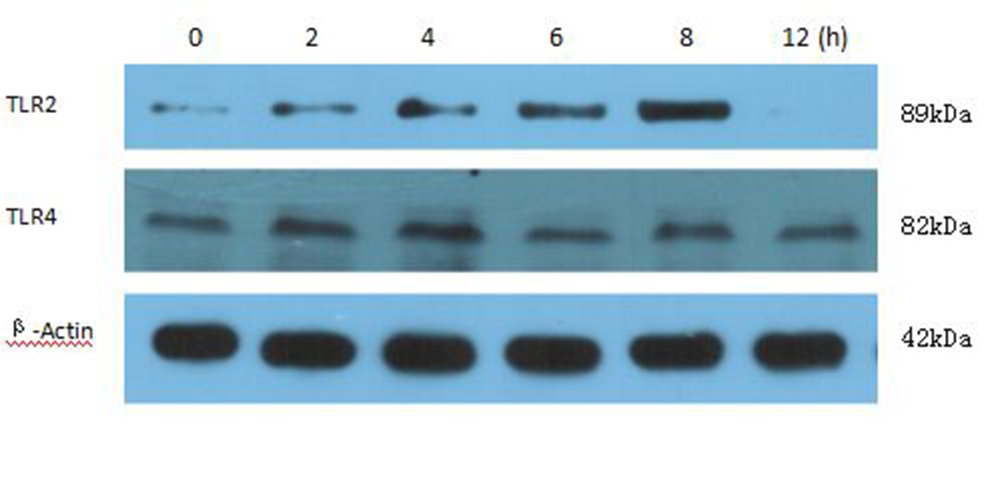

Supplement: Supplementary file 2 — Immunoblot of TLR2 and TLR4 expressed by AFs according to the time point. (TIF 266 kb) [file 12903_2018_656_MOESM2_ESM.tif]

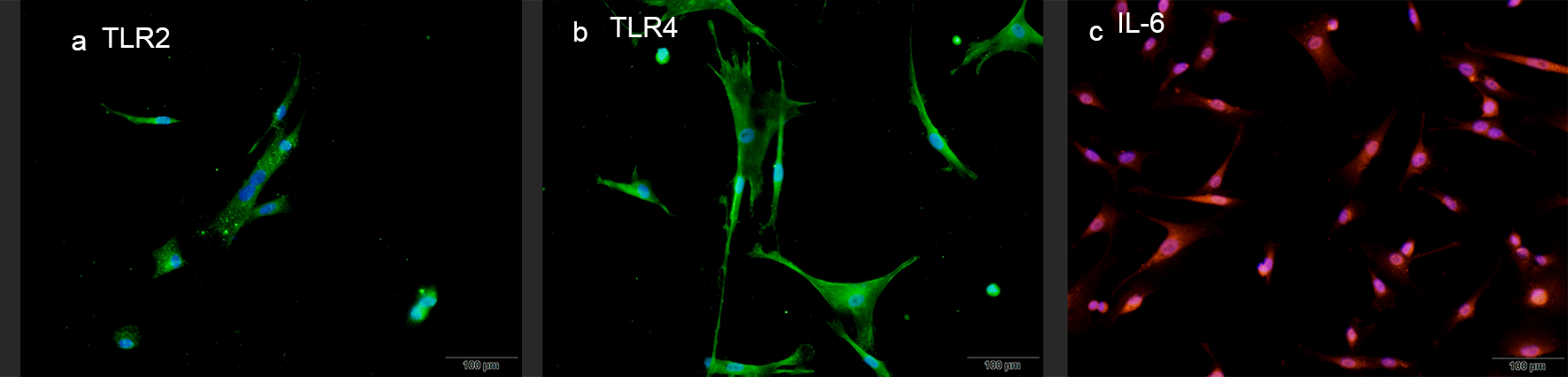

Supplement: Supplementary file 3 — Immunofluorescence of IL-6, TLR2, and TLR4 expressed by AFs after 8 h of pg.LPS stimulation. (TIF 274 kb) [file 12903_2018_656_MOESM3_ESM.tif]
